# Scrambling Eggs in Plastic Bottles

**DOI:** 10.1371/journal.pgen.0030006

**Published:** 2007-01-12

**Authors:** R. Scott Hawley, Dorothy Warburton

This issue of *PLoS Genetics* contains the second of two important papers describing the high levels of meiotic failure caused by exposure of female mice to a chemical known as Bisphenol A (BPA) [1]. In the first article [2], Pat Hunt and her collaborators demonstrated high levels of meiotic failure in females exposed to BPA, suggesting that BPA exposure affects maturing oocytes. This conclusion has been recently buttressed by an in vitro study by Can et al. [3] that demonstrates microtubule and centrosome changes in mouse eggs exposed to BPA, leading to an increase in aneuploidy. The new study takes this matter alarmingly further by demonstrating high levels of meiotic disruption, including perturbed synapsis and a greatly altered distribution of recombination events, in oocytes of fetuses being carried by mothers exposed to BPA. Part of the importance of these twin findings is that they provide people studying mammalian meiosis with powerful new tools for the study of the control of meiotic progression, both during and after meiotic prophase. Understanding the mode of action of BPA during both periods of the meiotic process may provide key insights into the regulation of meiosis.

But, perhaps more importantly, these observations represent the most convincing demonstration to date that environmental exposures may affect meiotic processes in mammals. Indeed, this discovery raises the troubling issue of whether or not this chemical, or other similar chemicals, pose a risk to meiotic fidelity in the human population, one that might increase the already high frequency of meiotic failures. As the authors point out, “We are exposed to BPA daily; it is a component of polycarbonate plastics, resins lining food/beverage containers, and additives in a variety of consumer products. More than 6 billion pounds are produced worldwide annually, and several studies have reported levels of BPA in human tissues in the parts per billion range.”

Mammalian oocytes enter meiotic prophase in the fetal ovary, where synapsis and recombination occur. Alteration in the number or distribution of recombination sites is well-known to play a major role in the origin of meiotic aneuploidy, especially in younger mothers. Thus, the findings in mice that BPA interferes with these processes raise the disturbing possibility that the exposure of women to BPA today might not be manifest for another generation. It is reasonable to ask whether the potential risks of ubiquitous exposure to BPA are sufficient to warrant the regulation of human exposure or at least to call for well-designed human studies.

Answering this question is not going to be easy for several reasons. First, when it comes to reproducing, we humans are terribly inefficient. No less than 15%–20% of human conceptions end in miscarriage, and an astonishing 50% of these are chromosomally abnormal. (Compare this to the common fruit fly, whose eggs are chromosomally normal in more than 99.9% of cases.) Against that sort of background of meiotic failure, it may be difficult indeed to identify *any* environmental component that influences the rate of nondisjunction. Another complication is that most chromosomally abnormal conceptions are lost before birth, so that studies of live births provide an infrequent and nonrepresentative sample of all aneuploid conceptions.

**Figure:**
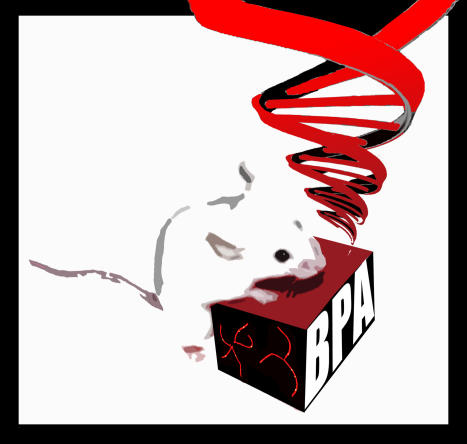
Studies of BPA Exposure in Mice Yield Key Information about Its Potential Effects on Meiosis (Image: Amy Vodicka, eStyle Graphics, http://www.estylegraphics.com)

Second, attempts to identify environmental factors associated with the frequency of chromosome errors have been almost uniformly negative [4]. Surveys of miscarriages in very different populations find little or no difference in the rates of trisomies, other than those that can be explained by differences in mean maternal age, suggesting that most environmental differences have at best a weak effect on the level of meiotic failure. Two major studies, one in New York City, which included many African-Americans and Hispanics [5], and one in Hawaii, which included many people of Asian and Hawaiian descent [6], gave results that were extremely similar in all respects. We can conclude that high levels of aneuploidy are a fact of human life that is independent of race and socioeconomic status, factors usually associated with environmental differences. The high rate of chromosomal anomalies in our species seems to be built into our biology and is not usually the result of the accumulation of adverse events.

Third, the strongest predictor of the frequency of meiotic error in oocytes is increasing maternal age. Simply put, as woman age from 25 to 45 their risk of producing an oocyte with an extra chromosome rises by more than 10-fold. Any attempt to identify an environmental influence on the rate of meiotic missegregation will need to be teased away from concomitant changes in this far more significant source of variation. We have witnessed a dramatic change in reproductive patterns in virtually a single generation. Reliable birth control methods have provided latitude, and many women are choosing to postpone childbearing for as long as possible. As more older women reproduce, the frequency of aneuploidy goes up. This can be best appreciated by comparing the data from two studies of human miscarriages conducted 20 years apart (see Figure 1). The proportion of miscarriages with trisomy has doubled, almost certainly because of the change in the maternal age distribution. This astoundingly strong maternal-age effect raises the possibility that any contribution of environmental agents to trisomy frequency pales beside elevated risk of aneuploidy caused by couples choosing to postpone childbearing till later in reproductive life.

**Figure 1:**
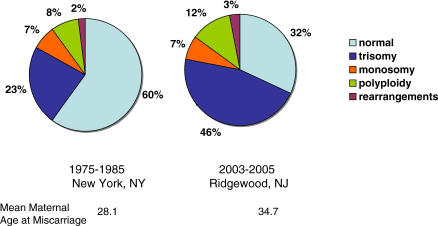
Effect of Maternal Age on the Distribution of Chromosome Anomalies in Miscarriages Distribution of chromosome abnormalities among consecutive karyotyped miscarriages with developmental age ≤18 weeks, from (left) New York City from 1974 to 1985 and (right) Ridgewood, New Jersey, from 2003 to 2005 (Warburton, Kline, and Kinney, unpublished data).

Fourth, Hunt and her collaborators have recently published an equally intriguing paper showing that a mutant in the mouse *Smc1β* gene produces an age-dependent increase in nondisjunction that parallels that seen in human females [7]. Thus, the rate of trisomy or susceptibility to aneugens may also be influenced by genetic variation in the human population. If this is indeed the case, it will seriously confound any effort to assess the effect of any given environmental component.

Fifth, and perhaps most critically, the BPA-induced damage to meiotic pairing and synapsis observed in mice by Hunt and her collaborators in the new study would not manifest itself for two generations. Thus, at least some of the effects of exposing a 26-year-old woman to chemicals such as BPA might take 20–30 years to manifest themselves in her grandchildren. A proper study of this problem would require assessing the woman's level of chemical exposure now and maintaining those data for two to three decades. It's a bit like wanting to do genetics on blue whales or giant sequoia trees; a curious problem, but not one most researchers will be willing to tackle.

Despite all these difficulties, there are nonetheless consistent rumblings that the fertility of our species is declining and that environmental exposures are at least partly to blame. BPA belongs to the class of endocrine disruptors that have received a lot of attention lately, but, in humans, these questions about chromosome damage remain in murky waters. Based on his observations in the Danish population, physician Niels Skakkebaek has championed the hypothesis that estrogenic exposures have resulted in a testicular dysgenesis syndrome in humans that includes a drop in sperm counts and an increase in testicular cancer and morphological aberrations of the external genitalia [8]. However, direct links are still lacking even in the male. What about the female? Is the incidence of female infertility increasing? The high intrinsic rate of aneuploidy and the changing reproductive patterns of human females will conspire against any researcher who endeavors to address the question of declining female fertility.

And yet, with all these caveats in place, and all those obvious difficulties in view, we still need to answer the question, “Are chemicals such as BPA aneugenic in humans?” It is obviously not going to be an easy question to answer, and even asking it is likely to create a storm of controversy, but it is also not a question that we have the luxury of ignoring—our fertility is fragile enough to require careful protection. We are convinced that the studies presented by Hunt and her collaborators will inspire scientists in both government and industry, who are far more skilled in toxicology than ourselves, to begin the serious task of addressing these questions. We argue that such toxicology-based questions might best be guided by the detailed molecular studies of BPA-induced meiotic failures in the mouse. 

## Abbreviations

BPA: Bisphenol A
